# Reduced Proteasome Degradation of HSF‐1 Shifts Protein Stress Management With Age in *Caenorhabditis elegans*

**DOI:** 10.1111/acel.70399

**Published:** 2026-01-29

**Authors:** Hongwei Wang, Fengzhen Sun, Zhidong He, Xiaojie Wang, Hao Liu, Mengjiao Song, Qingxia Chen, Zhixue Li, Ligang Wu, Xiumin Yan, Xueliang Zhu, Yidong Shen

**Affiliations:** ^1^ State Key Laboratory of Cell Biology, Shanghai Institute of Biochemistry and Cell Biology, Center for Excellence in Molecular Cell Science Chinese Academy of Sciences Shanghai China; ^2^ University of Chinese Academy of Sciences Beijing China; ^3^ Shanghai Institute of Immunity and Infection, Chinese Academy of Sciences Shanghai China; ^4^ Ministry of Education‐Shanghai Key Laboratory of Children's Environmental Health, Institute of Early Life Health, Xinhua Hospital Shanghai Jiao Tong University School of Medicine Shanghai China

**Keywords:** HSF‐1, proteasome, stress resistance

## Abstract

To maintain protein homeostasis, which is essential for health, animals have developed complex protective mechanisms against various acute and chronic stresses. However, the coordination of responses to these protein stresses, especially their age‐dependent changes, is not well understood. HSF‐1 is a key regulator of protein homeostasis. Our study identifies PBS‐7, a proteasome subunit, as its crucial regulator. In aged *C. elegans* , decreased PBS‐7 binding reduces proteasome‐mediated degradation of HSF‐1. The increase in HSF‐1 enhances responses to chronic stresses, like accumulating protein aggregates, by upregulating heat shock proteins (HSPs) and autophagy genes. Meanwhile, the upregulated HSPs suppress the activation of HSF‐1 upon acute stress, such as heat shock. Our findings reveal a mechanism that coordinates responses to acute and chronic protein stresses and highlights an adaptation prioritising protection against increasing protein aggregates in ageing.

## Graphical abstract

Ageing *C. elegans* prioritises chronic protein stress protection over acute stress response by coordinating HSF‐1 activity. Decreased PBS‐7 binding in aged worms reduces HSF‐1 degradation, enhancing its ability to upregulate protective factors against accumulating protein aggregates while simultaneously suppressing its activation under acute heat shock.

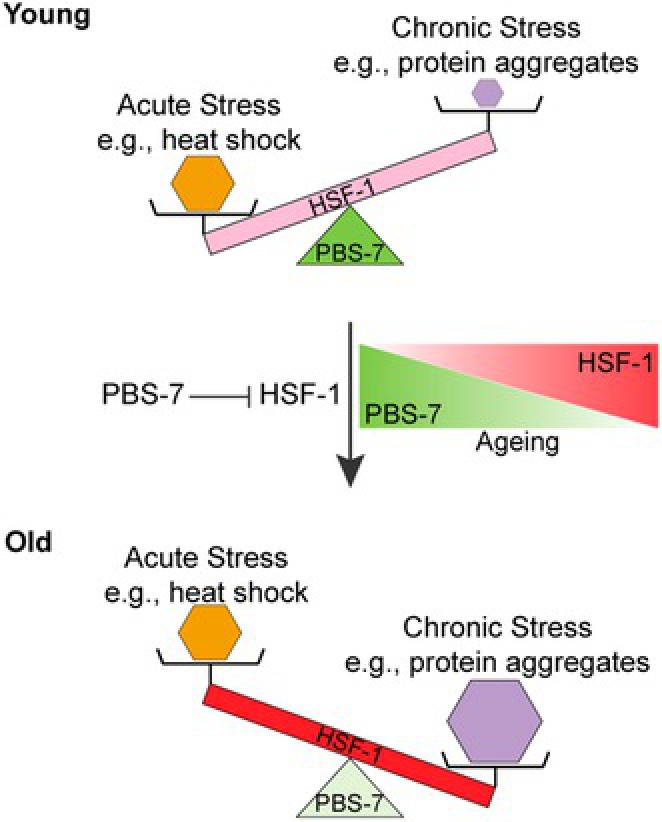

**Abbreviations**
*C. elegans*
: *Caenorhabditis elegans*
CMD‐1: calmodulin 1
HSE: heat shock elements
HSF‐1: heat shock factor protein 1
HSP: heat shock protein
MosSCI: Mos1‐mediated Single Copy Insertion
PBS‐7: proteasome subunit beta 7
PolyQ: polyglutamine
PSM: peptide‐spectrum match
PSMB4: proteasome subunit beta type‐4
RNAi RNA: interference
UPS: ubiquitin‐proteasome system

Protein homeostasis is essential to health and related to various disorders. In living organisms, cells are under constant toxic stresses, upsetting the balance of protein metabolism (protein stresses). By the temporal scale, protein stresses are categorised into acute and chronic ones. The most representative and well‐studied acute protein stress is heat shock. The abrupt increase of environmental temperature disrupts protein folding, causing denaturation and aggregation of proteins. The long‐term accumulation of damaged protein is a prevalent chronic protein stress and is considered to be the primary cause of various neurodegenerative diseases (Hoppe and Cohen 2020).

In response to persistent protein stresses, cells evolve a sophisticated protein quality control system, with the conserved transcription factor, heat shock factor 1 (HSF1), standing at its centre (Akerfelt et al. 2010; Hoppe and Cohen 2020). Upon stress, HSF1 dissociates from the heat shock protein (HSP) complex, trimerises, binds to the heat shock elements (HSEs) in HSP genes, and drives their expression (Akerfelt et al. 2010). Functioning as molecular chaperones, HSPs recognise misfolded proteins or protein aggregates, leading to their repair or degradation. Of note, HSPs and HSF1 form a feedback loop, keeping a fine‐tuning and quick response to various stresses (Akerfelt et al. 2010). In addition to HSPs, HSF1 controls other targets critical to protein homeostasis, such as autophagy and the ubiquitin‐proteasome system (Kinet et al. 2016; Kovacs et al. 2019; Kumsta et al. 2017; Li et al. 2017; Watanabe et al. 2017; Zhou et al. 2019).

Despite the protection by this elaborate protein quality system, the cellular resistances to both acute and chronic protein stresses decline with ageing, making aged animals vulnerable to abrupt environmental shifts (e.g., heat shock) and susceptible to a series of disorders (e.g., Alzheimer's disease) (Hartl 2016). The deregulation of HSF1 is considered to play a key role in the age‐dependent reduction of protein stress (Ben‐Zvi et al. 2009). Consistently, HSF1 is required in various longevity models for their improved protein homeostasis (Cohen et al. 2006; Hsu et al. 2003; Morley and Morimoto 2004; Morphis et al. 2022; Shemesh et al. 2013; Steinkraus et al. 2008; Walker and Lithgow 2003). However, the molecular mechanism underlying the age‐dependent dysregulation of HSF1 remains poorly understood.

In this study, we identified the proteasome‐mediated degradation controlled by *pbs‐7*, a proteasome subunit, as a critical regulation of HSF‐1 in ageing *Caenorhabditis elegans* . The reduced PBS‐7‐HSF‐1 interaction downregulates HSF‐1 degradation in aged worms, upregulating *hsp* genes and autophagy against the accumulating protein aggregates, whereas blunting the reaction of HSF‐1 to heat shock. Our results thus show an age‐dependent shift of the HSF‐1‐centred protein quality control system from acute to chronic protein stress, highlighting an adaptation to prioritise protection against the increasing protein aggregates in aged animals.

## 1 Results

### 1.1 The Activation of HSF‐1 Upon Heat Shock Is Reduced in Aged Worms

**FIGURE 1:**
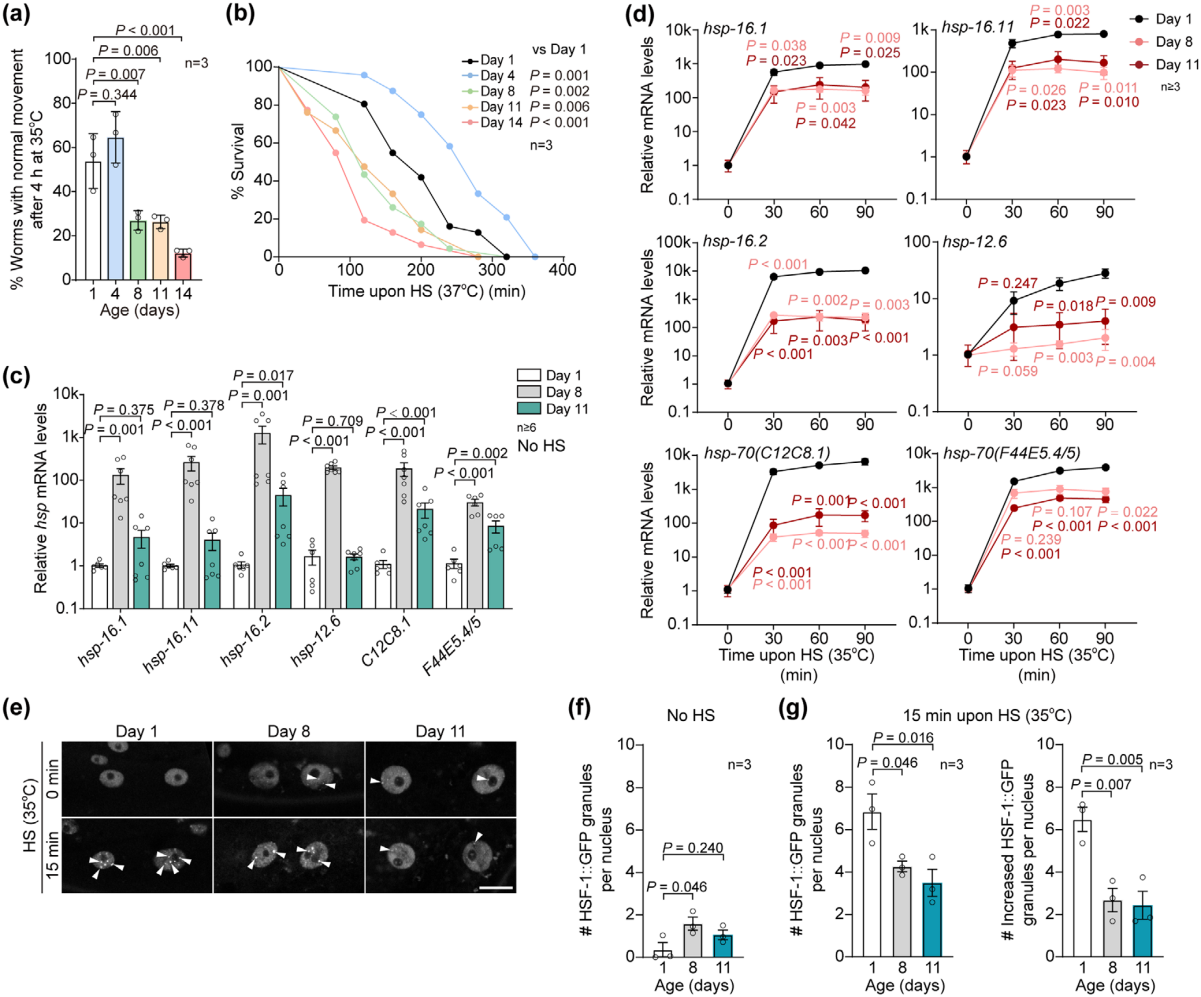
The heat shock response decreases in aged worms. (a) The recovery rate of wild‐type worms at indicated ages after a 4‐h heat shock at 35°C. 3 biological replicates were examined. (b) The survival curves of worms at indicated ages cultured upon a heat shock (HS) of 37°C for each age; more than 60 worms in three biological replicates were examined. (c) qPCR‐measured mRNA levels of indicated *hsp* genes in young and aged worms at Day 1, 8, and 11 of adulthood. 6–7 biological replicates were examined. (d) The induction of *hsp* genes upon heat shock is reduced in aged worms. Worms at Day 1, 8, and 11 of adulthood were treated with a heat shock of 35°C for the indicated time and then collected for RT‐qPCR analysis. The *hsps* levels at 0 min post‐HS are set as 1. 3–7 biological replicates were examined. Error bars: SEM. (e) The nuclear stress granules of HSF‐1::GFP (arrowheads) in the worm hypodermis at indicated ages with or without heat shock. Scale bar: 10 μm. (f) The number of HSF‐1::GFP nuclear stress granules in the worms without heat shock More than 50 worms in three replicates were examined. (g) The induction of HSF‐1::GFP nuclear stress granules upon heat shock decreases with ageing. More than 50 worms in three replicates were examined. One‐way ANOVA with Tukey's multiple comparison test in (a, f, and g), two‐way ANOVA with Tukey's multiple comparison test in (c and d), Gehan‐Breslow‐Wilcoxon test in (b).

*C. elegans* is regularly cultured at 20°C. To trace the change of heat resistance during ageing, we examined worms at Day 1, 4, 8, 11, and 14 of adulthood for their recovery rates following a 4‐h heat shock of 35°C and their survival at 37°C. Consistent with previous reports (Bansal et al. 2015), worms' resistance to acute heat stress declines with ageing (Figure 1a,b). RNAi against *hsf‐1* further impaired the remaining resistance to heat stress in the worms at Day 11 of adulthood (Figure S1a,b), confirming its reported role in heat shock responses (Akerfelt et al. 2010; McMillan et al. 1998; Zhang et al. 2002).

To monitor the change in HSF‐1 activation, we next examined the induction of *hsp* genes upon heat shock in worms of various ages. To our surprise, all examined *hsp* genes exhibited elevated expression in ageing when worms are cultured at regular temperature (Figure 1c). In contrast, their induction upon heat shock was remarkably reduced in aged worms at Day 11 of adulthood (Figure 1d and Figure S1c). HSF‐1 forms subnuclear puncta known as nuclear stress granule to drive *hsps* expression (Cotto et al. 1997; Morton and Lamitina 2013; Zhang et al. 2022). Similar to our observations of *hsps* expression, we found more subnuclear puncta of endogenously GFP‐tagged HSF‐1 (hereafter referred to as HSF‐1::GFP), which are reported as stress granules critical to HSF‐1 activity (Cotto et al. 1997; Morton and Lamitina 2013; Zhang et al. 2022), in the hypodermis of aged worms without heat shock (Figure 1e,f) and an age‐dependent reduction in the increase of these puncta upon heat shock (Figure 1e,g). Therefore, HSF‐1 has a higher basal activity but a dulled response to acute heat stress in aged worms.

### 1.2 Proteosome‐Mediated HSF‐1 Degradation Decreases With Ageing

As we previously reported (Zhou et al. 2019), the protein level of HSF‐1::GFP increases in aged worms (Figure S2a), potentially causing its elevated basal activity. Unlike the elevated protein level, the mRNA expression of *hsf‐1* remained unchanged during ageing (Figure S2b). So, the age‐dependent increase of HSF‐1 protein could be due to post‐translational regulation.

**FIGURE 2:**
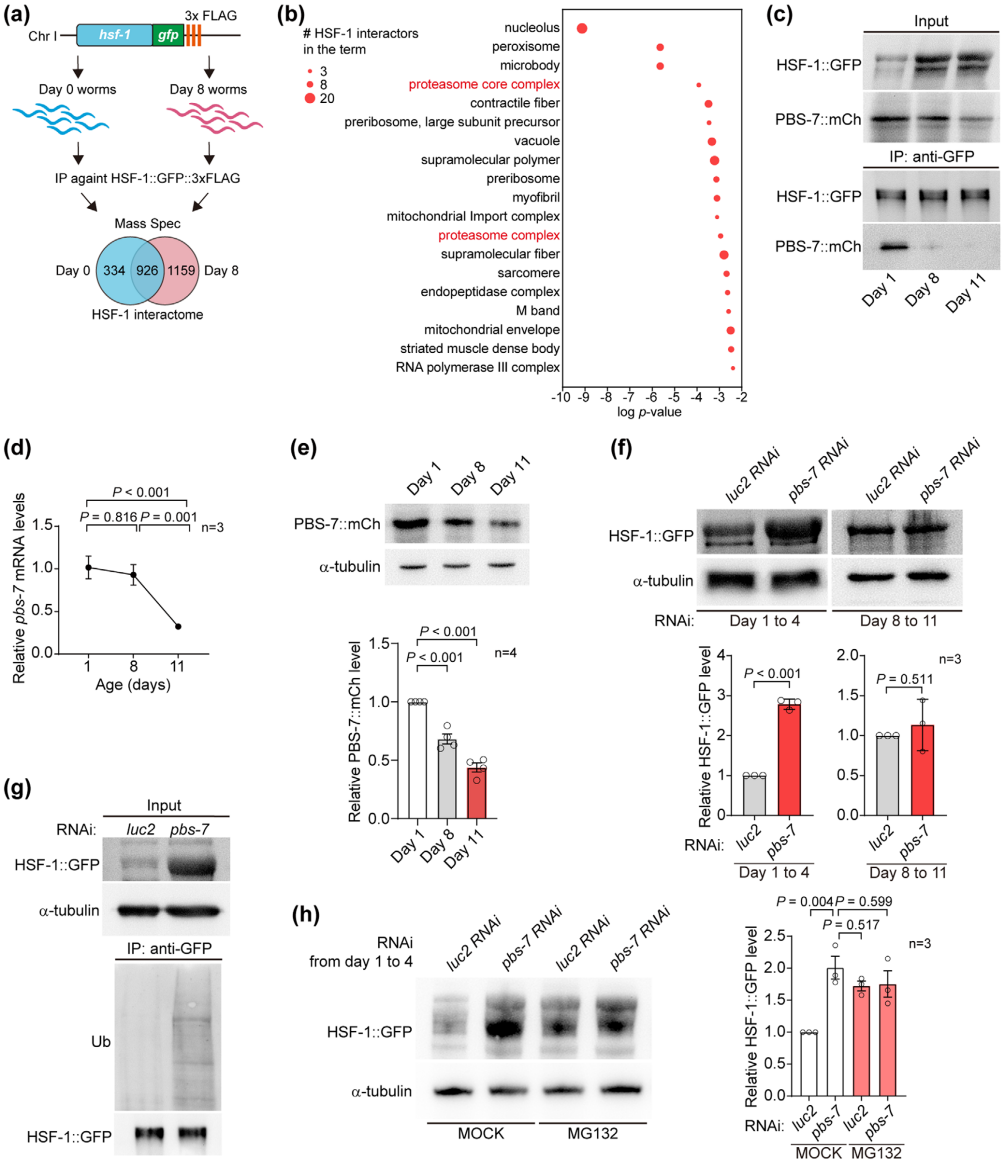
The proteasome‐mediated degradation of HSF‐1 decreases with ageing. (a) The analysis of age‐dependent HSF‐1 interactomes. 334 and 1159 proteins were identified as HSF‐1 interactors specific in young (Day 0) and aged (Day 8) worms, respectively. See Table S1 for the detailed interactomic data. (b) The cellular components (CC) terms in the gene set enrichment analysis of HSF‐1 interactors specific to young worms. Also see Table S2. (c) Co‐immunoprecipitation of mCherry (mCh) tagged PBS‐7 with HSF‐1::GFP in the worms of indicated ages. PBS‐7::mCh is expressed from a single copy transgene, *pbs‐7p::pbs‐7::mCh*, inserted at *ttTi5605* by CRISPR‐Cas9. HSF‐1::GFP is from the endogenously *gfp*‐tagged *hsf‐1*. (d) RT‐qPCR of *pbs‐7* in the worms of indicated ages. 3 biological replicates were examined. (e) PBS‐7::mCh is downregulated with ageing. 4 biological replicates were examined. (f) RNAi against *pbs‐7* in young worms but not in aged worms upregulates HSF‐1::GFP. 3 biological replicates were examined. (g) Inhibiting *pbs‐7* in young worms increases the ubiquitination of HSF‐1::GFP. (h) *pbs‐7* RNAi in young worms upregulates HSF‐1::GFP through proteasome. MG132 is a proteasome inhibitor. 3 biological replicates were examined. *luc2* is a luciferase gene, serving as the negative control of RNAi. α‐tubulin serves as the loading control in (e, f, g, and h). HSF‐1::GFP serves as the loading control of immunoprecipitants in (c and g). Hypergeometric test in (a), one‐way ANOVA with Tukey's multiple comparison test in (d, e, and h), unpaired *t*‐test in (f).

We then examined the interactome of HSF‐1::GFP in young adult worms without bearing fertilised eggs (Day 0 of adulthood) and post‐reproductive aged worms (Day 8 of adulthood) (Figure 2a). Worms were thoroughly lysed to detect both cytosolic and nuclear interactors of HSF‐1::GFP. Reported HSF‐1 interactors, such as HSP‐70s and CMD‐1 (Masser et al. 2019; Shen et al. 2008; Shi et al. 1998), were detected in the co‐immunoprecipitants with HSF‐1::GFP by mass spectrometry (Table S1), validating our interactomic analysis. GSEA analysis showed an enrichment of ‘proteosome’ related proteins in the Day 0‐specific HSF‐1 interactome (Figure 2b, Table S2). Among the identified proteosome components in HSF‐1 interactome, PBS‐7, the proteosome subunit and the worm ortholog of mammalian PSMB4, showed the highest affinity by sum PEP score (Figure S3a, Table S1). In HEK293T cells, GFP‐HSF‐1 also co‐immunoprecipitated with FLAG‐PBS‐7 (Figure S3b). To examine the interaction between PBS‐7 and HSF‐1 in vivo, we made a MosSCI insertion of a transgene expressing PBS‐7::mCherry by its native promoter at *ttTi5605* location and crossed it with the strain expressing HSF‐1::GFP. Consistent with our mass spectrometry data, PBS‐7::mCherry exhibited a clear interaction with HSF‐1::GFP in young worms at Day 1 of adulthood, whereas the level of PBS‐7 interacting with HSF‐1::GFP decreased in aged worms at Day 8 of adulthood (Figure 2c).

qPCR of worms at different ages showed an age‐dependent decline of *pbs‐7* (Figure 2d). Similarly, a previous proteomics analysis found PBS‐7 decreased with age (Figure S3c) (Walther et al. 2015). We also found the protein level of PBS‐7::mCherry decreased in aged worms (Figure 2e). Therefore, the downregulation of PBS‐7 expression likely contributes to the reduced PBS‐7‐HSF‐1 interaction in aged worms.

The interaction between HSF‐1 and PBS‐7 suggests that it could be degraded by the ubiquitin‐proteasome system (UPS). Indeed, inhibiting *pbs‐7* in young adults by RNAi from Day 1 to 4 of adulthood increased the protein level of HSF‐1 (Figure 2f, Figure S3d). Moreover, purified HSF‐1 from the worms upon *pbs‐7* RNAi showed a stronger ubiquitination level (Figure 2g). In young worms, inhibiting proteasome with MG132 upregulated HSF‐1 in a similar manner as *pbs‐7* RNAi, whereas *pbs‐7* RNAi did not further increase HSF‐1 in worms treated with MG132 (Figure 2h), confirming that PBS‐7 induces HSF‐1 degradation through UPS. Blocking autophagy using chloroquine did not block the pbs‐7 RNAi‐induced upregulation of HSF‐1 (Figure S3e), indicating that PBS‐7 does not regulate HSF‐1 through autophagy. Since the interaction between HSF‐1::GFP with PBS‐7::mCherry decreased in aged worms (Figure 2c), the age‐dependent increase of HSF‐1 is likely due to its reduced proteasome‐mediated degradation. If so, suppressing *pbs‐7* in aged worms should have little effect on HSF‐1 level because the HSF‐1‐PBS‐7 interaction is already reduced. In line with this speculation, *pbs‐7* RNAi treatment in aged worms from Day 8 to 11 of adulthood did not change HSF‐1 level (Figure 2f).

### 1.3 The Reduced HSF‐1 Degradation Improves Resistance to Protein Aggregates

**FIGURE 3:**
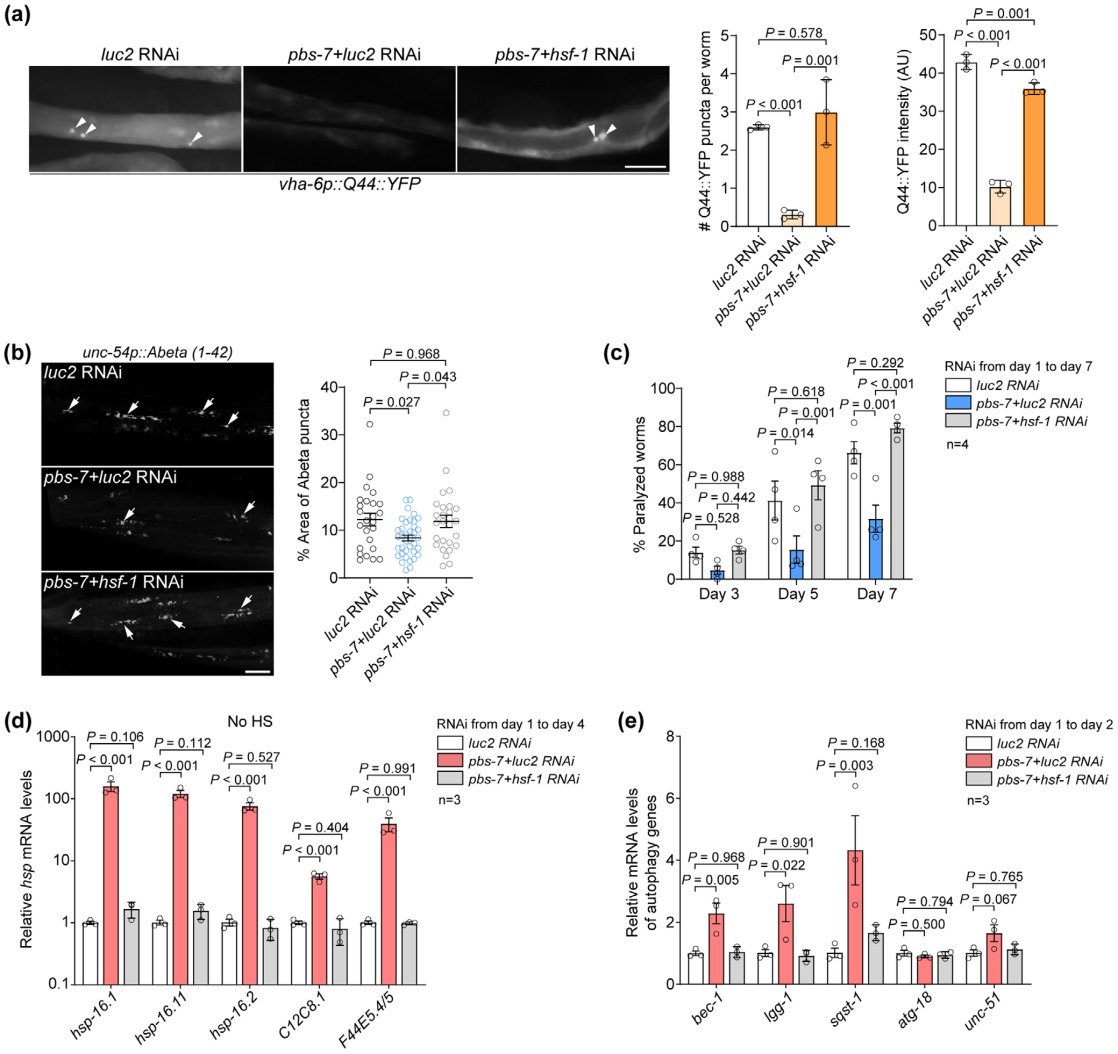
The reduction of proteasome‐mediated HSF‐1 degradation improves resistance to chronic protein stress. (a) RNAi against *pbs‐7* reduces polyQ expression and the resulting aggregates (arrowheads) through *hsf‐1*. Scale bar: 50 μm. More than 50 worms in three biological replicates were scored in each sample. (b) Suppressing *pbs‐7* decreases human Aβ (1–42) aggregates (arrows) in the body wall muscle through *hsf‐1*. Scale bar: 10 μm. Error bars: SEM. More than 25 worms in three biological replicates were examined in each sample. (c) The paralysis rate of the worms expressing human Aβ (1–42) in the body wall muscle upon indicated RNAi treatments. More than 175 worms in four biological replicates were examined in each sample. (d) The mRNA levels of *hsp* genes upon indicated RNAi treatments in young worms. (e) *pbs‐7* RNAi in young worms upregulates autophagy genes through *hsf‐1*. 3 biological replicates were examined in (d and e). Two‐way ANOVA with Tukey's multiple comparison test in (c), one‐way ANOVA with Tukey's multiple comparison test in the rest.

During ageing, worms suffer chronic protein stress from accumulating protein aggregates (Walther et al. 2015). The increased HSF‐1 in aged worms could be adaptive to this chronic protein stress. We tested this hypothesis using the worms expressing polyglutamine (polyQ) in the intestine. RNAi against *pbs‐7* (Figure S3d), which is to mimic the age‐dependent reduction of PBS‐7 levels and UPS‐mediated degradation of HSF‐1 (Figure 2), remarkably reduced polyQ expression and the resulting aggregates in worms at Day 5 of adulthood (Figure 3a, Figure S3f). A further suppression of *hsf‐1* restored the polyQ expression and aggregates in *pbs‐7* RNAi‐treated worms (Figure 3a, Figure S3g). We further examined the worms expressing human Aβ (1–42) in body wall muscle. Similarly, *pbs‐7* RNAi reduced Aβ aggregates, whereas a co‐suppression of *pbs‐7* and *hsf‐1* did not (Figure 3b). Moreover, inhibiting *pbs‐7* improved the health of the Aβ disease model worms through *hsf‐1*, as measured by the paralysis rate (Figure 3c). Therefore, the reduction of PBS‐7 promotes protein homeostasis through upregulating HSF‐1.

HSF‐1 controls multiple pathways against chronic protein stress (Kumsta et al. 2017; Seo et al. 2013; Watanabe et al. 2017; Williams et al. 2020). As expected, RNAi against *pbs‐7* in young adult worms induces the transcription of various *hsps* and a series of autophagy genes (Figure 3d,e). Double RNAi against *pbs‐7* and *hsf‐1* reduced the expression of these genes to the same level as that in worms treated with *luc2* RNAi (Figure 3d,e). We further used the mCherry::GFP::LGG‐1 reporter to examine autophagy in worms treated with *pbs‐7* RNAi (Zhang et al. 2015) (Figure S4a). Consistent with the upregulation of autophagy genes, suppressing *pbs‐7* increased autophagosomes and autolysosomes in the body wall muscle (Figure S4b,c). Taken together, the upregulation of HSF‐1 via UPS protects worms against chronic protein stress through increased autophagy and chaperone proteins.

### 1.4 The Reduced HSF‐1 Degradation Suppresses Heat Shock Responses

The activation of HSF‐1 upon heat shock requires the dissociation from its bound HSPs (Akerfelt et al. 2010). Therefore, the elevated basal level of *HSPs* expression could upregulate the HSP‐HSF‐1 interaction upon heat shock and, in turn, impair heat resistance in aged worms. Consistently, our mass spectrometry data found 7 HSPs specifically in the co‐immunoprecipitants of HSF‐1::GFP from aged worms (Figure S5a, Table S1). Among them is one HSP70 (F44E5.4/F44E5.5), which is reported to suppress HSF‐1 transcriptional activity (Abravaya et al. 1992; Baler et al. 1992; Kmiecik et al. 2020; Shi et al. 1998). *F44E5.4* and *F44E5.5* are duplicates of each other, located closely on the same chromosome and sharing promoter regions with HSF‐1 binding sites (Araya et al. 2014) (Figure S5b). In this study, we do not distinguish these two genes and refer to them as *F44E5.4/5*. We further found that HA‐F44E5.4/5 interacted with GFP‐HSF‐1 in HEK293T cells (Figure S5c).

**FIGURE 4:**
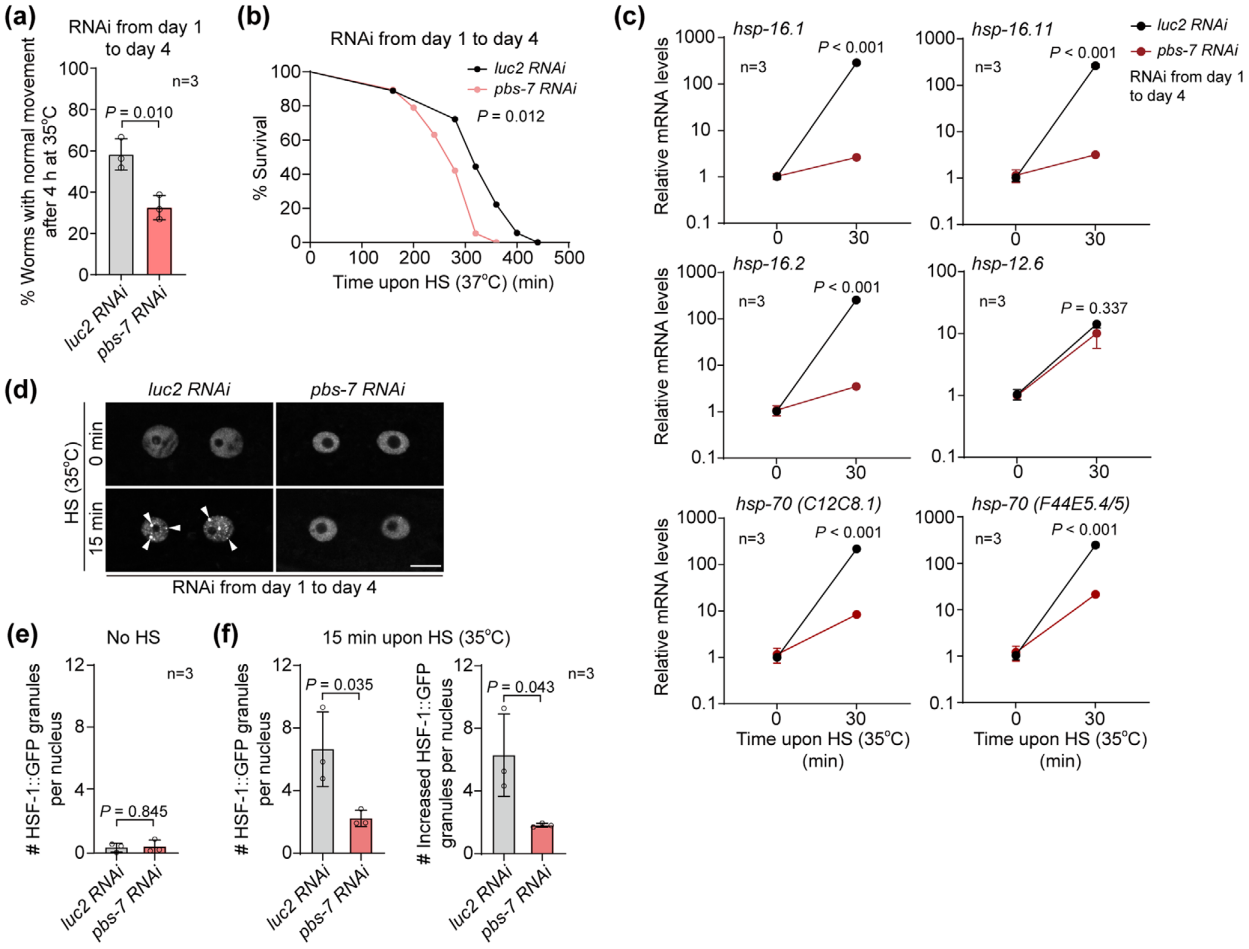
The heat shock response is impaired by the reduced HSF‐1 degradation. (a) The recovery rate of young worms treated with indicated RNAi after a 4‐h heat shock of 35°C. 3 biological replicates were examined. (b) The survival rate of young worms treated with indicated RNAi upon a heat shock of 37°C. More than 50 worms in 3 biological replicates were examined for each treatment. (c) *pbs‐7* RNAi in young worms suppresses the induction of *hsp* genes upon heat shock (HS) of 35°C. 3 biological replicates were examined. (d–f) Suppressing *pbs‐7* in young worms reduces the increase of nuclear stress granules of HSF‐1::GFP (arrowheads) in young worms. Scale bar: 10 μm. For each treatment, more than 50 worms from 3 biological replicates were examined. Unpaired *t*‐test in (a, c, e, and f), Gehan‐Breslow‐Wilcoxon test in (b).

To further pursue this hypothesis, we treated young adult worms with *pbs‐7* RNAi from Day 1 to 4 of adulthood to mimic the increased HSF‐1 and *hsps* levels in aged worms (Figures 2 and 3). As we expected, inhibiting *pbs‐7* impaired heat resistance, reducing the recovery rate post heat shock and the survival rate upon heat stress (Figure 4a,b). Consistently, treating young worms with *pbs‐7* RNAi suppressed the induction of *hsp* genes and HSF‐1::GFP granules in nuclei upon heat shock (Figure 4c–f).

**FIGURE 5:**
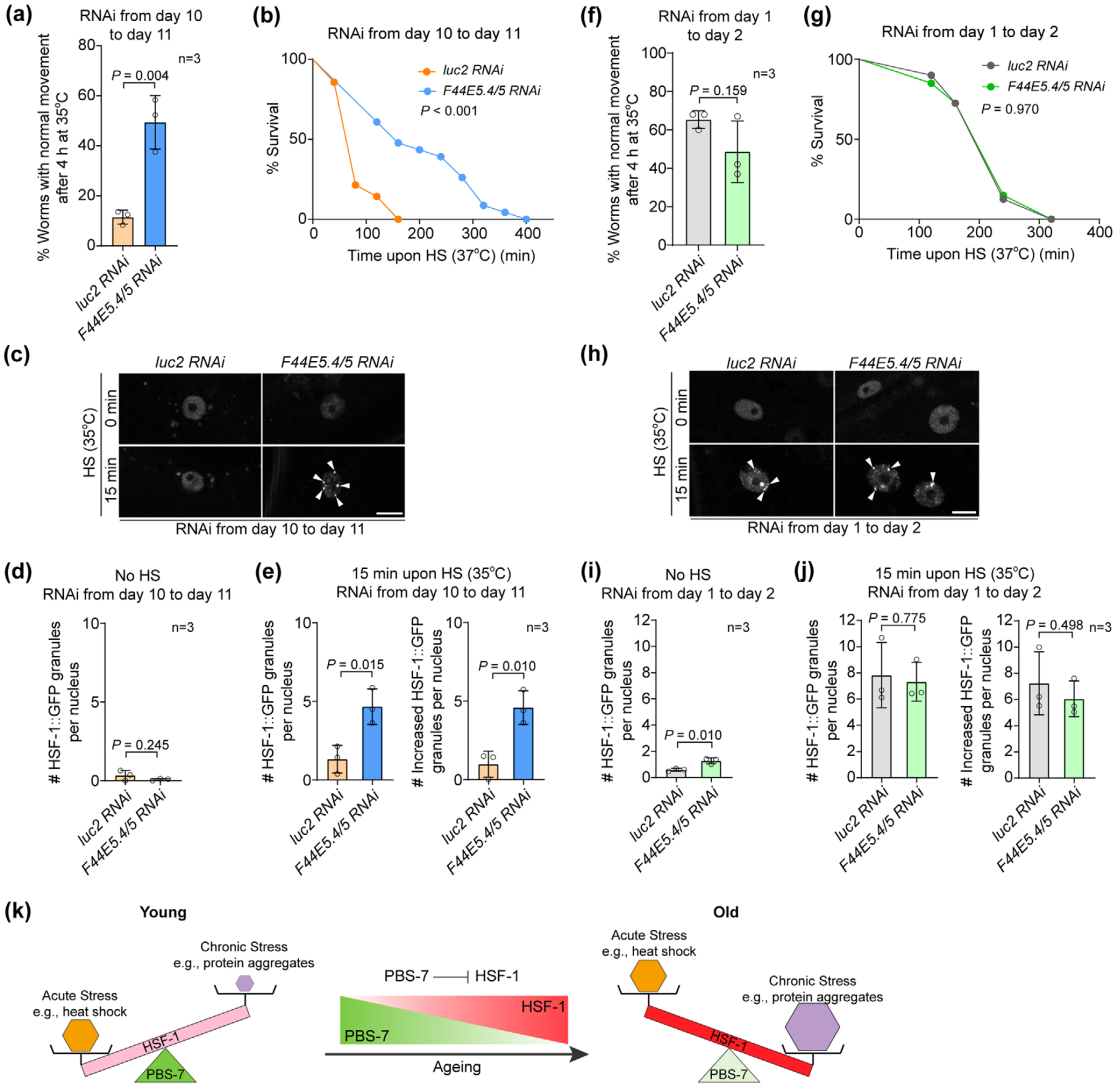
Suppressing *F44E5.4/5* in aged worms promotes heat stress resistance. (a) *F44E5.4/5* RNAi in aged worms increases the recovery rate post a 4 h heat shock at 35°C. (b) Inhibiting *F44E5.4/5* in aged worms promotes survival upon heat stress at 37°C. (c–e) *F44E5.4/5* regulates the induction of nuclear stress granules of HSF‐1::GFP (arrowheads) in aged worms upon heat shock of 35°C. Scale bar: 10 μm. (f, g) Suppressing *F44E5.4/5* in young worms does not affect the recovery rate post a 4 h heat shock at 35°C (f) or the survival upon heat stress at 37°C (g). (h–j) *F44E5.4/5* does not regulate the induction of nuclear stress granules of HSF‐1::GFP (arrowheads) in young worms upon heat shock of 35°C. Scale bar: 5 μm. (k) The trade‐off of HSF‐1 activity as an adaptation to ageing. The decline in PBS‐7‐mediated regulation of HSF‐1 during ageing leads to an increase in HSF‐1 protein levels and shifts its primary role from responding to acute stress, such as heat shock, to addressing chronic stress, such as protein aggregation. Please see discussion for more information. 3 biological replicates were examined in (a and f). In (b and g), more than 48 worms in 3 biological replicates were examined for each treatment. In (d, e, i, and j), for each treatment, more than 50 worms from 3 biological replicates were examined. Unpaired *t*‐test in (a, d, e, f, i, and j), Gehan‐Breslow‐Wilcoxon test in (b and g).

If the elevated basal level of *hsps* blunts HSF‐1 activation upon heat shock in aged worms, a proper inhibition of *hsp* should improve the worms' heat stress resistance. We then treated aged worms with a mild *F44E5.4/5* RNAi by ~40% from Day 10 to 11 (Figure S6a), trying to mimic its level in young worms. In line with our hypothesis, the mild reduction of *F44E5.4/5* in aged worms improved the recovery rate post heat shock and the survival upon heat stress (Figure 5a,b). Moreover, the inhibition of *F44E5.4/5* in aged worms also increased the nuclear stress granules of HSF‐1::GFP upon heat shock (Figure 5c–e). To confirm that the improved response to heat stress is due to the suppression of the age‐dependent increase of *F44E5.4/5*, we next treated young worms with the same *F44E5.4/5* RNAi. As expected, suppressing *F44E5.4/5* in young worms did not affect heat stress resistance (Figure 5f,g) or the formation of HSF‐1::GFP stress granules in the nucleus (Figure 5h–j). Meanwhile, RNAi against another two *hsps*, *C12C8.1* and *hsp‐90*, showed little effects on the heat resistance of aged worms (Figure S6b–d), indicating not all *hsps* are involved in the age‐dependent regulation of HSF‐1. Taken together, the proteasome‐mediated HSF‐1 degradation is critical for worms' resistance to acute heat stress.

## 2 Discussion

HSF1 is a master regulator of protein homeostasis across organisms. While imbalanced protein metabolism is a hallmark of ageing (Lopez‐Otin et al. 2023), the changes of HSF1 in ageing remain poorly understood. A previous study highlighted altered DNA methylation of *hsp* genes in early adulthood as a key regulator of HSF‐1 activity during worm ageing (Labbadia and Morimoto 2015).

In this study, we identified the UPS pathway as another age‐dependent regulator of HSF‐1, directly influencing its protein level. By examining its interactome in young and aged worms, we found that the interaction between HSF‐1 and proteasome components, particularly PBS‐7, declines with age, resulting in reduced proteasome‐mediated degradation of HSF‐1 (Figure 2). It is reported that the ubiquitinated proteome undergoes significant changes during *C. elegans* ageing (Koyuncu et al. 2021). Thus, in addition to the decrease of PBS‐7 in ageing (Figure 2), alterations in HSF‐1 ubiquitination could also contribute to its reduced UPS‐mediated degradation.

UPS‐mediated regulation may enable HSF‐1 to sense the deteriorating proteostasis and serve as an adaptative mechanism during ageing. The UPS itself is essential to maintain protein homeostasis by removing damaged or misfolded proteins, and its dysregulation is a major contributor to impaired proteostasis in ageing (Koyuncu et al. 2021; Lopez‐Otin et al. 2023; Walther et al. 2015). The PBS‐7‐HSF‐1 interaction could serve as a checkpoint for ageing adaptation. Upon the weakening of UPS in aged animals, the PBS‐7‐HSF‐1 interaction is impaired, causing the upregulation of HSF‐1 to mitigate the disrupted proteostasis (Figure 5k). Since we also identified other proteasome components in the HSF‐1 interactome, this checkpoint could involve HSF‐1 interaction with a wider range of proteasome proteins. Besides, accumulated damaged proteins could also contribute to HSF‐1 activation with ageing. In line with this speculation, we observed an upregulation of HSF‐1 protein (Zhou et al. 2019) (Figure 2), increased HSF‐1 nuclear granules, and elevated *hsps* transcription (Figure 1) in aged WT worms without heat shock. Consistently, upregulating HSF‐1 in young disease model worms by *pbs‐7* RNAi reduced polyglutamine and amyloid‐beta aggregation, reducing paralysis (Figure 3).

HSF‐1 forms stress granules in nuclei (Cotto et al. 1997; Morton and Lamitina 2013; Zhang et al. 2022), and drives multiple protein homeostasis pathways, including the induction of *hsp* chaperones that assist in protein folding and the refolding of misfolded or denatured proteins, as well as autophagy that clears unwanted proteins (Kumsta et al. 2017; Li et al. 2017; Watanabe et al. 2017). Consistent with the age‐dependent increase of HSF‐1, we and others observed a significant increase of *hsps* transcription in aged worms (Liang et al. 2014) (Figure 1). Furthermore, increasing HSF‐1 in young worms also induces *hsps* and autophagy as we speculated (Figure 3, Figure S4). Together, these findings suggest that the upregulation of HSF‐1 protects against chronic protein stress in aged worms.

Interestingly, we found that increased basal levels of *hsps* in aged worms impair heat shock response rather than enhance it. Upon heat shock, HSPs dissociate from HSF‐1, allowing HSF‐1 to activate its downstream transcriptional program for the heat shock responses (Akerfelt et al. 2010). The elevated basal level of *hsps* in unstressed aged worms may suppress the release of HSPs from HSF‐1 during heat stress, thereby reducing heat shock resistance. As we hypothesised, mass spectrometry analysis showed HSF‐1 has a stronger interaction with HSPs in aged worms (Figure S5). The induction of HSF‐1 activity is also significantly weaker in aged worms or in young worms with elevated HSF‐1 (Figures 1 and 4). Consistently, a mild inhibition of *hsp* (e.g., *F44E5.4/5*) in aged worms improves HSF‐1 activation (Figure 5). Of note, suppressing the other two *hsps*, *C12C8.1* and *hsp‐90*, in aged worms barely changed heat resistance (Figure S6), indicating that not all *hsps* contribute to the age‐dependent regulation of HSF‐1. These findings suggest that UPS‐mediated regulation finely tunes HSF‐1 activities in response to various protein stresses.

During ageing, there is a marked change in protein stress. Young organisms are primarily challenged by acute stressors, such as sudden temperature changes, which require a rapid and robust activation of HSF1. In contrast, ageing is characterised by the gradual accumulation of misfolded and aggregated proteins, introducing an additional chronic proteotoxic stress that becomes the dire challenge in older animals (Lopez‐Otin et al. 2023). This age‐dependent change in protein stress necessitates an increased basal activation of HSF1 to alleviate the persistent proteotoxic burden, albeit at the cost of a diminished response to heat shock (Figure 5k). Our findings of the switch of HSF1 activity to chronic stress highlight a critical aspect of adaptive ageing, where cellular machinery evolves to prioritise long‐term maintenance over immediate stress responses.

## 3 Methods

### 3.1 *C. elegans* Strains and Culture

*C. elegans* strains used in this study are listed in Table S3. *C. elegans* were grown with standard techniques at 20°C (Brenner 1974), unless otherwise noted. Some strains were provided by the CGC, which is funded by the NIH Office of Research Infrastructure Programs (P40 OD010440).

To collect worms at different ages, worms were synchronised by collecting eggs and transferred to fresh plates every 2 days in adulthood to avoid contamination from progenies. Dead worms and worms undergoing internal hatching, bursting vulva, crawling off the plates, or contamination were discarded.

To treat worms with 20 μM MG132 or 50 μM chloroquine, 200 μL of 1 mM MG132 or 2.5 mM chloroquine was spread onto seeded 6‐cm plates. Worms were transferred onto these plates the next day.

### 3.2 Plasmid Construction

To generate L4440::F44E5.4/5, *F44E5.4/5* cDNA was PCR amplified from N2 cDNA and cloned into L4440.

To generate HA‐F44E5.4/5, *F44E5.4/5* cDNA was amplified from N2 cDNA with HA in the primer and cloned into pcDNA3.1.

To generate GFP‐HSF‐1, *hsf‐1* cDNA was amplified from N2 cDNA and cloned into pEGFP‐c1.

To generate FLAG‐PBS‐7, *pbs‐7* cDNA was amplified from N2 cDNA with 3xFLAG in the primer and cloned into pEGFP‐c1 to replace the EGFP.

For genome editing by CRISPR‐Cas9, plasmids were constructed following the report from Goldstein lab (Dickinson et al. 2015). To generate pCZGY2728*‐Ppbs‐7::pbs‐7::mCherry*, 310 bp of *pbs‐7* promoter and *pbs‐7* gDNA were amplified from the genomic DNA of N2, and inserted into pCZGY2728 (a gift from Zhiping Wang lab) with mCherry, using NEBuilder HiFi DNA Assembly Master Mix (New England Biolabs). Target sites in the templates were modified with synonymous mutations.

All plasmids have been submitted to BRICS (http://www.brics.ac.cn/plasmid/template/article/about.html), with the identifiers of SP‐3220, SP‐3221, SP‐3222, SP‐3223, and SP‐3224.

### 3.3 Transgenes

To generate *syd144[ttTi5605‐Ppbs‐7::pbs‐7::mCherry]*, pDD122 (a gift from Zhiping Wang lab) (50 ng/μL) and pCZGY2728*‐Ppbs‐7::pbs‐7::mCherry* (10 ng/μL) were coinjected into N2 with an injection marker of *myo‐2p::gfp* (2 ng/μL). Screening of integrated strain is as reported (Dickinson et al. 2015).

### 3.4 RNA Interference

Worms were grown on HT115 expressing dsRNA against indicated genes during the corresponding time. HT115 expressing dsRNA against *luc2* (a real gene but irrelevant to worm biology) served as a control, which was a gift from Adam Antebi's lab (MPI‐AGE). The RNAi clones of sjj_Y53C10A.12 and sjj_F39H1.5, respectively targeting *hsf‐1* and *pbs‐7*, are gifts from Shiqing Cai's lab (ION, CAS).

### 3.5 Heat Resistance Assays

For thermorecovery assay, synchronised worms on plates were incubated at 35°C for 4 h. Following heat shock treatment, worms were incubated at 20°C to recover for 48 h. After recovery, worms moving normally in response to gentle prodding were scored as ‘normal’, whereas worms exhibiting paralysis, uncoordinated movement, lethargy, or irresponsiveness to prodding were scored as ‘abnormal’. Three biological replicates with at least 50 worms in each were performed.

For heat‐resistant assay, worms were incubated at 37°C. Worm survival was examined every 40 min during heat shock until all worms were dead. Three biological replicates with at least 50 worms in each were performed.

### 3.6 Microscopy

For microscopy, worms placed on 5% agar pads were anesthetised using 10 mM levamisole. Worms were imaged on an Olympus BX53 microscope for PolyQ aggregates or on a Zeiss LSM880 for the rest of the assays. Fluorescence intensity was measured by Adobe Photoshop or ImageJ.

### 3.7 Analysis of Stress Granule

Stress granules in hypodermal cell nuclei were counted in worms with or without a 15 min heat shock at 35°C. HSF‐1::GFP puncta with a size bigger than 5 pixels and an intensity stronger than 10,000 AU were defined as stress granules. The average brightness in the hypodermal cell nuclei is below 5000 AU in the corresponding images. Three biological replicates with at least 50 worms in each were performed.

### 3.8 Analysis of Protein Aggregates

The numbers of intestinal polyQ aggregates in OG412 worms under corresponding RNAi were counted at indicated days of adulthood using Imaris 9.7.1 (Oxford Instruments). At least 50 worms from three biological replicates were scored in each sample. For Aβ aggregates, CL2006 expressing human Aβ (1–42) in body wall muscle (BWM) was treated with corresponding RNAi from Day 1 to 3 of adulthood and stained using 1 mM X‐34 in 10 mM Tris (pH 8.0) for 2 h at RT (Link et al. 2001). The stained worms were then transferred onto OP50 plates, allowed to destain overnight, and imaged on the next day. In each myocyte, one confocal optical slice where the striation and the aggregates were clear was examined. ImageJ was used to threshold Aβ aggregates and measure their area. The area of myocyte was measured by manually outlining the cell with ImageJ.

### 3.9 Autophagy Analysis

Autophagy in BWM was measured using mCherry::GFP::LGG‐1 reporter (Zhou et al. 2019). In brief, each BWM cell was scored for mCherry::GFP::LGG‐1 puncta from one confocal optical slice where the striation was clear. The puncta in a 100 μm^2^ area were counted using ComDet v.0.5.1 in ImageJ. The number of autophagosomes was calculated as the mCherry/GFP‐positive puncta, and the number of autolysosomes as (the total number of mCherry‐positive puncta—the mCherry/GFP‐positive puncta).

### 3.10 Paralysis Analysis

Paralysed CL2006 worms were scored as worms unable to move (roll their whole body) upon prodding. Dead worms and worms undergoing internal hatching, bursting vulva, or crawling off the plates were censored.

### 3.11 Quantitative RT‐PCR

More than 100 synchronised worms were collected into QIAzol reagent (QIAGEN) in each sample. Total RNA was then column purified by SteadyPure Universal RNA Extraction Kit (AG) and subjected to cDNA preparation by Evo M‐MLV RT Premix for qPCR (AG), following the manufacturer's instructions. Quantitative RT‐PCR was performed with 2xNovoStart SYBR qPCR SuperMix Plus (Novoprotein) on a CFX384 TouchTM Real‐Time PCR Detection System (Bio‐Rad). A combination of mRNA levels of *ama‐1* and *cdc‐42* was used for normalisation. At least three biological repeats were tested. Primer sequences are listed in Table S3 or as reported (Kumsta et al. 2017; Xu et al. 2019).

### 3.12 Cell Culture and Transfection

HEK293T cells (ATCC) were maintained in DMEM medium supplemented with 10% fetal bovine serum (Cellmax) at 37°C, 5% CO_2_. Cells were authenticated by morphology and tested for mycoplasma contamination prior to experiments.

HEK293T cells were transfected using PEI (Proteintech) following the manufacturer's instructions. When testing the interaction of HSF‐1 with PBS‐7 or F44E5.4/5, cells in each well of a 6‐well plate were transfected with 3 μg of GFP‐HSF‐1 and 4 μg of FLAG‐PBS‐7 or HA‐F44E5.4/5. Cells were collected 48 h post transfection.

### 3.13 Immunoblot

Synchronised worms at corresponding ages were collected in M9. To harvest worms at Day 8 and 11 of adulthood, ~80 synchronised worms were collected for each sample. Worms were lysed by 4× SDS loading buffer (TAKARA, Cat#9173) after three times of freezing and thawing with liquid nitrogen.

Cells were collected in RIPA buffer (Beyotime) supplemented with Protease Inhibitor Cocktail Set III (Merck), NaF, NaVO_4_, and PMSF (Roche) and lysed by pulse sonication on a Scientz‐IID ultrasonic homogeniser (SCIENTZ). To detect ubiquitylation in lysates, 5 mM N‐ethylmaleimide (NEM) was added to the lysis buffer prior to use. The protein concentrations of lysates were measured using the BCA Protein Assay Kit (Beyotime).

Proteins were separated by reducing SDS‐PAGE and transferred to nitrocellulose filter membranes. Membranes were then blotted with primary antibodies against GFP (1:1000 dilution, Cat# sc‐9996, Santa Cruz), GFP (1:2000 dilution, Cat# 50430‐2‐AP, Proteintech), FLAG (1:2000 dilution, Cat# F7425, Sigma‐Aldrich), mCherry (1:2000 dilution, Cat# NBP1‐96752, Novus Biologicals), mCherry (1:2000 dilution, Cat# ab167453, Abcam), HA (1:2000 dilution, Cat# ab137838, Abcam), Ubiquitin (1:2000 dilution, Cat# 10201‐2‐AP, Proteintech), α‐tubulin (1:2000 dilution, Cat# T5168, Sigma‐Aldrich), and secondary antibodies against Rabbit IgG (1:5000 dilution, Cat# G‐21234, Thermo Fisher Scientific) or Mouse IgG (1:5000 dilution, Cat# G‐21040, Thermo Fisher Scientific). Signals of western blotting were measured using Adobe Photoshop. Background signals were subtracted as reported (Shen et al. 2012).

### 3.14 Immunoprecipitation and Mass Spectrometry

For immunoprecipitation of proteins from HEK293T cells, cells were lysed using Cell lysis buffer for western and IP (Beyotime) supplemented with PMSF (Roche).

For immunoprecipitation of proteins from *C. elegans* , synchronised worms were grown in liquid culture fed with HB101 until the desired ages. Pelleted worms were washed three to five times with M9 until the supernatant was clear. Afterwards, worms were washed with ddH_2_O. Worm pellets were resuspended in an equal volume of ice‐cold 2× lysis buffer (60 mM HEPES, pH 7.4, 100 mM KCl, 4 mM MgCl_2_, 0.1% Triton X, 10% Glycerol, 2 mM DTT) supplemented with Protease Inhibitor Cocktail Set III (Merck), NaF, NaVO_4_, and PMSF (Roche). Worms were homogenised with 0.7 mm and 1 mm Zirconia/Silica Beads (BioSpec) on an OMNI Bead Ruptor 12 (OMNI). After a subsequent centrifugation, the supernatant was collected as worm lysate.

Cell or worm lysate was incubated with Anti‐GFP Affinity Beads 4FF or Anti‐DYKDDDDK Affinity Beads (Smart‐Lifesciences) at 4°C for 4 h and then washed four times with wash buffer (30 mM HEPES, pH 7.4, 100 mM KCl, 0.1% Triton X‐100, 2 mM MgCl_2_, 10% Glycerol, 1 mM DTT). The immunoprecipitants were eluted using SDS loading buffer by boiling for western blot. For mass spectrometry analysis of HSF‐1 interactors, the immunoprecipitants were precipitated with acetone. The protein pellet was dried using a Speedvac for 1–2 min. The pellet was subsequently dissolved in 8 M urea, 100 mM Tris–HCl, pH 8.5. TCEP (final concentration 5 mM) (Thermo Scientific) and N‐ethylmaleimide (final concentration 10 mM) (Sigma) for reduction and alkylation were added to the solution and incubated at room temperature for 30 min, respectively. The protein mixture was diluted four times and digested overnight with Trypsin at 1:50 (w/w) (Promega). The digested peptide solutions were desalted using a MonoSpinTM C18 column (GL Science, Tokyo, Japan) and dried with a SpeedVac. The peptide mixture was loaded onto a homemade 15 cm‐long pulled‐tip analytical column (Aqua, C18, 750 μm OD× 360 μm ID, 3 μm particle size, 125 Å pore diameter, Phenomenex, Torrance, CA) connected to an Easy‐nLC 1000 nano HPLC (Thermo Scientific, San Jose, CA) for shotgun mass spectrometry analysis. Proteins with non‐zero PSM (peptide‐spectrum match) in any of the three biological replicates were kept as HSF‐1 interactors for analysis. Because we focused on interactors specifically identified in young or aged worms, no further signal normalisation was performed.

### 3.15 Statistics

Results are presented as mean ± SD unless otherwise noted. n refers to the number of biological replicates. Statistical tests were performed as indicated using GraphPad Prism (GraphPad software). Statistical significance was defined as a *p* value smaller than 0.05. Note that dCt or ddCt was used for all the statistical analyses of the qPCR assays. All analyses were blinded. The detailed statistical information is shown in Table S4.

## Author Contributions

Hongwei Wang, Fengzhen Sun, Zhidong He, and Yidong Shen conceived the project and designed the experiments. Hongwei Wang, Fengzhen Sun, Zhidong He, and Xiaojie Wang performed experiments and analysed data, with the assistance of Mengjiao Song and Hao Liu helped in sample preparation for Mass‐Spec under the supervision of Xueliang Zhu and Qingxia Chen helped in co‐IP under the supervision of Xiumin Yan and Zhixue Li helped in bioinformatics under the supervision of Ligang Wu, Hongwei Wang, Fengzhen Sun, and Yidong Shen wrote the manuscript. All authors contributed to manuscript editing.

## Funding

This research was supported by the State Key Laboratory of Cell Biology, CAS.

## Conflicts of Interest

The authors declare no conflicts of interest.

## Supporting information


**Figure S1:** HSF‐1 is required for heat resistance in aged worms. (a) The recovery rate of worms at Day 11 of adulthood post the indicated time of heat shock at 35°C. Unpaired *t*‐test. 3 biological replicates were examined. (b) The survival curves of worms at Day 8 of adulthood with indicated RNAi treatment upon heat stress at 37°C. Gehan‐Breslow‐Wilcoxon test. More than 77 worms in three biological replicates were examined. (c) The mRNA levels of indicated *hsps* upon heat shock of 35°C. The *hsps* levels in worms at Day 1 of adulthood at 0 min post‐HS are set as 1. Error bars: SEM. Two‐way ANOVA with Tukey's multiple comparison test. 3–7 biological replicates were examined.

**Figure S2:** The age‐dependent changes in HSF‐1 expression. (a) HSF‐1::GFP is upregulated with ageing. α‐tubulin serves as the loading control. (b) The mRNA levels of *hsf‐1* in wild‐type worms at indicated ages. 3 biological replicates were examined. One‐way ANOVA with Tukey's multiple comparison test.

**Figure S3:** The interaction between PBS‐7 and HSF‐1 decreases with ageing. (a) PBS‐7 shows the highest confidence as HSF‐1::GFP interactor among the four proteosome components specifically detected in HSF‐1::GFP immunoprecipitants of young worms. Sum PEP (posterior error probability) Score is a metric to assess the confidence of an identified protein. The higher it is, the higher the confidence. (b) The co‐immunoprecipitation of FLAG‐PBS‐7 with GFP‐HSF‐1 in HEK293T cells. Representative blots from 3 biological replicates were shown. (c) Quantitative proteomic analysis shows an age‐dependent decrease of PBS‐7. The level of PBS‐7 at Day 1 of adulthood is set as 1. Data are from Walther et al. 2015. (d) RNAi efficiency of indicated *pbs‐7* RNAi treatments. PBS‐7::mCh is expressed from a single copy transgene, *pbs‐7p::pbs‐7::mCh*, inserted at *ttTi5605* by CRISPR‐Cas9. Representative blots from 3 biological replicates were shown. (e) Chloroquine treatment does not block the upregulation of HSF‐1::GFP upon *pbs‐7* RNAi in young worms. (f) The protein level of Q44::YFP upon indicated RNAi treatment. (g) *hsf‐1* RNAi efficiently knocks down the mRNA levels of *hsf‐1*. α‐tubulin serves as the loading control in (d, e, and g). Unpaired *t*‐test. 3 biological replicates were examined in (b–g). Error bars: SEM.

**Figure S4:** Suppressing *pbs‐7* improves autophagy in the body wall muscle. (a) A depiction of the analysis of autophagosome (AP) and autolysosome (AL) using the mCherry::GFP::LGG‐1 reporter. The acidic environment in AL quenches GFP fluorescence, turning the reporter into red. (b and c) Worms carrying the mCherry::GFP::LGG‐1 reporter were treated with indicated RNAi from Day 1 to 4 (b) or Day 9 (c) of adulthood. APs and ALs were scored in the body wall muscle. Arrows: AP, arrowheads: AL. Scale bars: 10 μm. Unpaired *t*‐test. More than 77 worms in three biological replicates were examined.

**Figure S5:** The age‐dependent HSF‐1 interactions with heat shock proteins. (a) The detected heat shock proteins (HSPs) in the co‐immunoprecipitants of HSF‐1::GFP by mass‐spectrometry. PSM, peptide‐spectrum matches, was used as a benchmark for the interaction with HSF‐1::GFP. Those specifically found from worms at Day 8 of adulthood are highlighted in red. (b) A depiction of F44E5.4 and F44E5.5 in the genome. The two genes are identical in cDNA sequences and located closely on Chromosome II. By ChIP‐Seq datasets in modENCODE, HSF‐1 binds to their shared promoter region. (c) HA‐F44E5.4/5 interacts with GFP‐HSF‐1 in HEK293T cells. Representative blots from 3 biological replicates were shown.

**Figure S6:** RNAi against *C12C8.1* or *hsp‐90* in aged worms barely improved heat resistance. (a) RNAi efficiency of *F44E5.4/5* RNAi. The worms expressing *F44E5.4/5::mCherry* in the intestine were treated with indicated RNAi and examined by western blot. α‐tubulin serves as the loading control. 3 biological replicates were examined. (b) RNAi against *C12C8.1* and *hsp‐90* efficiently suppressed target genes mRNA levels. 3 or 4 biological replicates were examined. (c and d) *C12C8.1* or *hsp‐90* RNAi in aged worms had little effect on worms recovery post 4‐h heat shock at 35°C (c) or the survival upon a 37°C heat shock (HS) (d). More than 60 worms in three biological replicates were examined. Unpaired *t*‐test in (a–c), Gehan‐Breslow‐Wilcoxon test in (d).

**Table S1:** HSF‐1::GFP interactors at Day 0 and 8 of adulthood by mass spectrometry.

**Table S2:** GSEA analysis of HSF‐1::GFP interactors specific in young and aged worms.

**Table S3:** The list of strains and oligos used in this study.

**Table S4:** The detailed statistics of the assays in this study.

## Data Availability

Source data are provided in this paper. Any remaining data that support the results of this study are available from the corresponding author upon reasonable request.
