# Supplementary figures and images for: Reduced Proteasome Degradation of HSF‐1 Shifts Protein Stress Management With Age in Caenorhabditis elegans
Source: Aging Cell. 2026 Jan 29;25(2):e70399. doi: 10.1111/acel.70399 (PMC12856053; doi:10.1111/acel.70399)

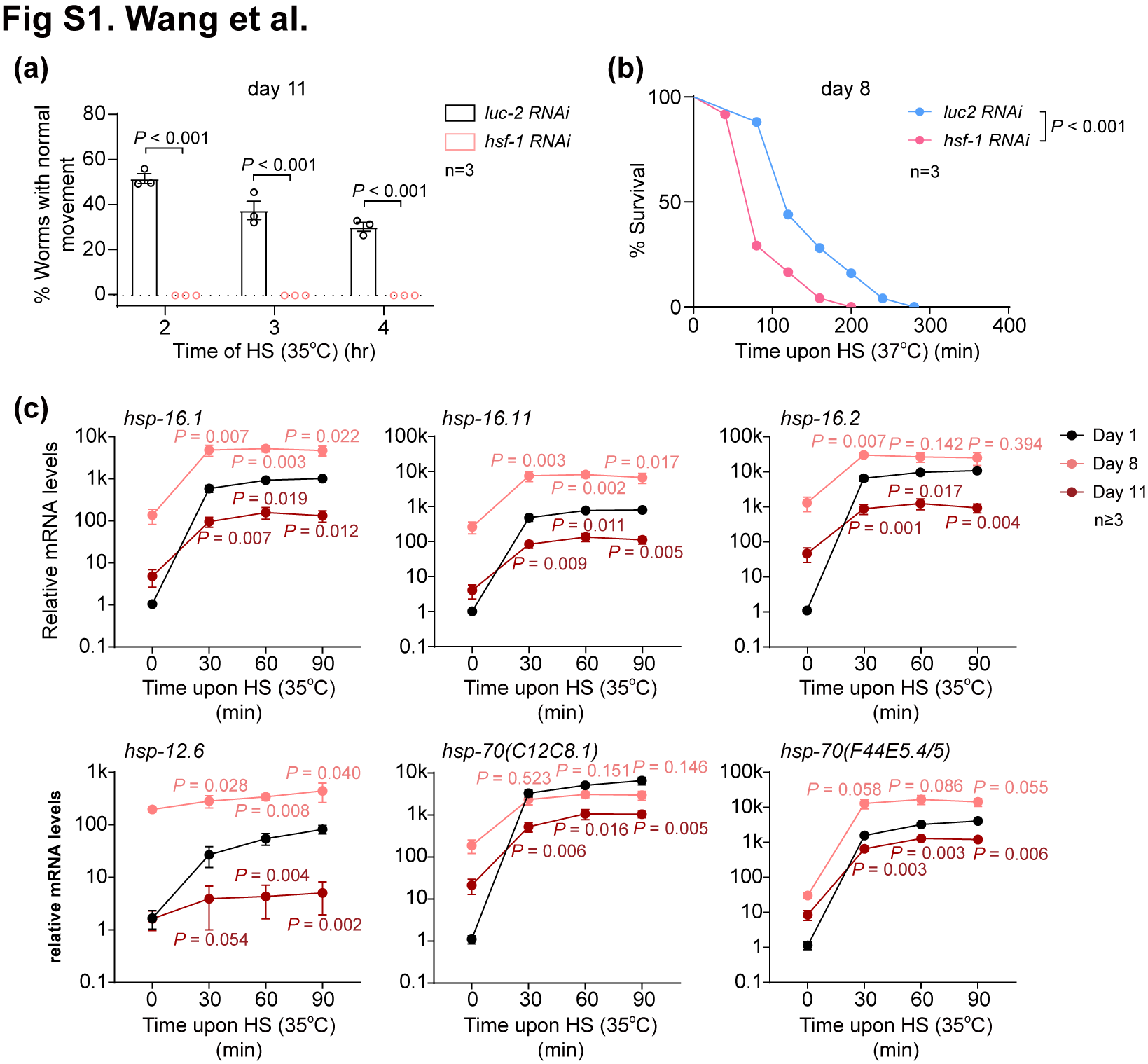

Supplement: Supplementary file 1 — Figure S1: HSF‐1 is required for heat resistance in aged worms. (a) The recovery rate of worms at Day 11 of adulthood post the indicated time of heat shock at 35°C. Unpaired t‐test. 3 biological replicates were examined. (b) The survival curves of worms at Day 8 of adulthood with indicated RNAi treatment upon heat stress at 37°C. Gehan‐Breslow‐Wilcoxon test. More than 77 worms in three biological replicates were examined. (c) The mRNA levels of indicated hsps upon heat shock of 35°C. The hsps levels in worms at Day 1 of adulthood at 0 min post‐HS are set as 1. Error bars: SEM. Two‐way ANOVA with Tukey's multiple comparison test. 3–7 biological replicates were examined. [file ACEL-25-e70399-s010.tif]

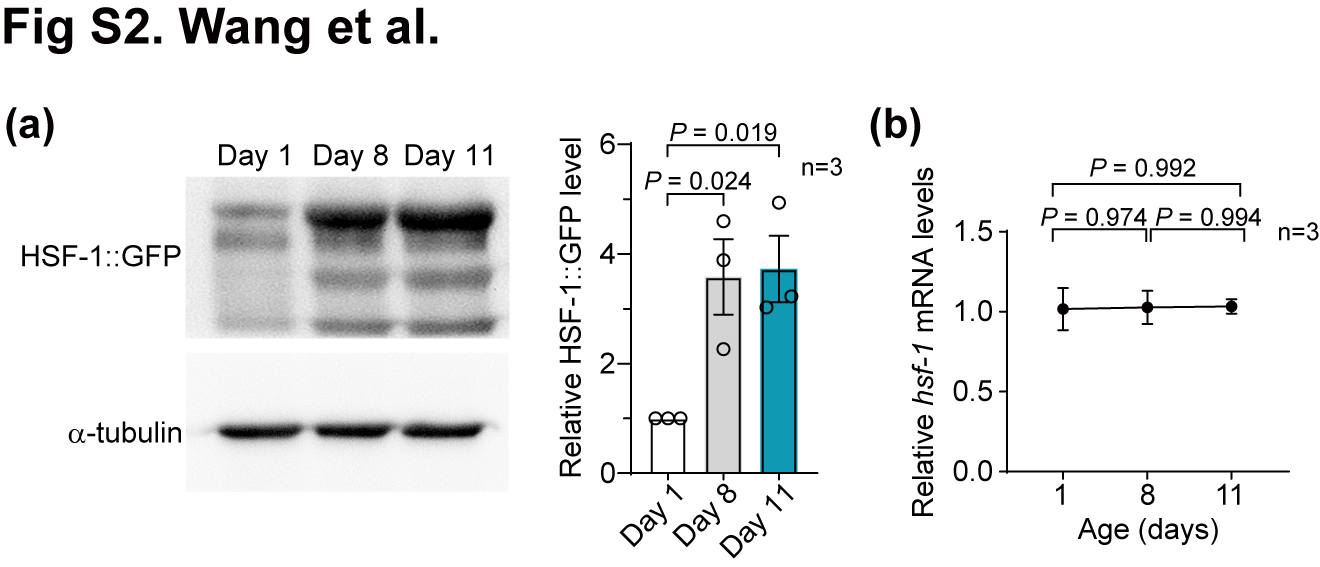

Supplement: Supplementary file 2 — Figure S2: The age‐dependent changes in HSF‐1 expression. (a) HSF‐1::GFP is upregulated with ageing. α‐tubulin serves as the loading control. (b) The mRNA levels of hsf‐1 in wild‐type worms at indicated ages. 3 biological replicates were examined. One‐way ANOVA with Tukey's multiple comparison test. [file ACEL-25-e70399-s009.tif]

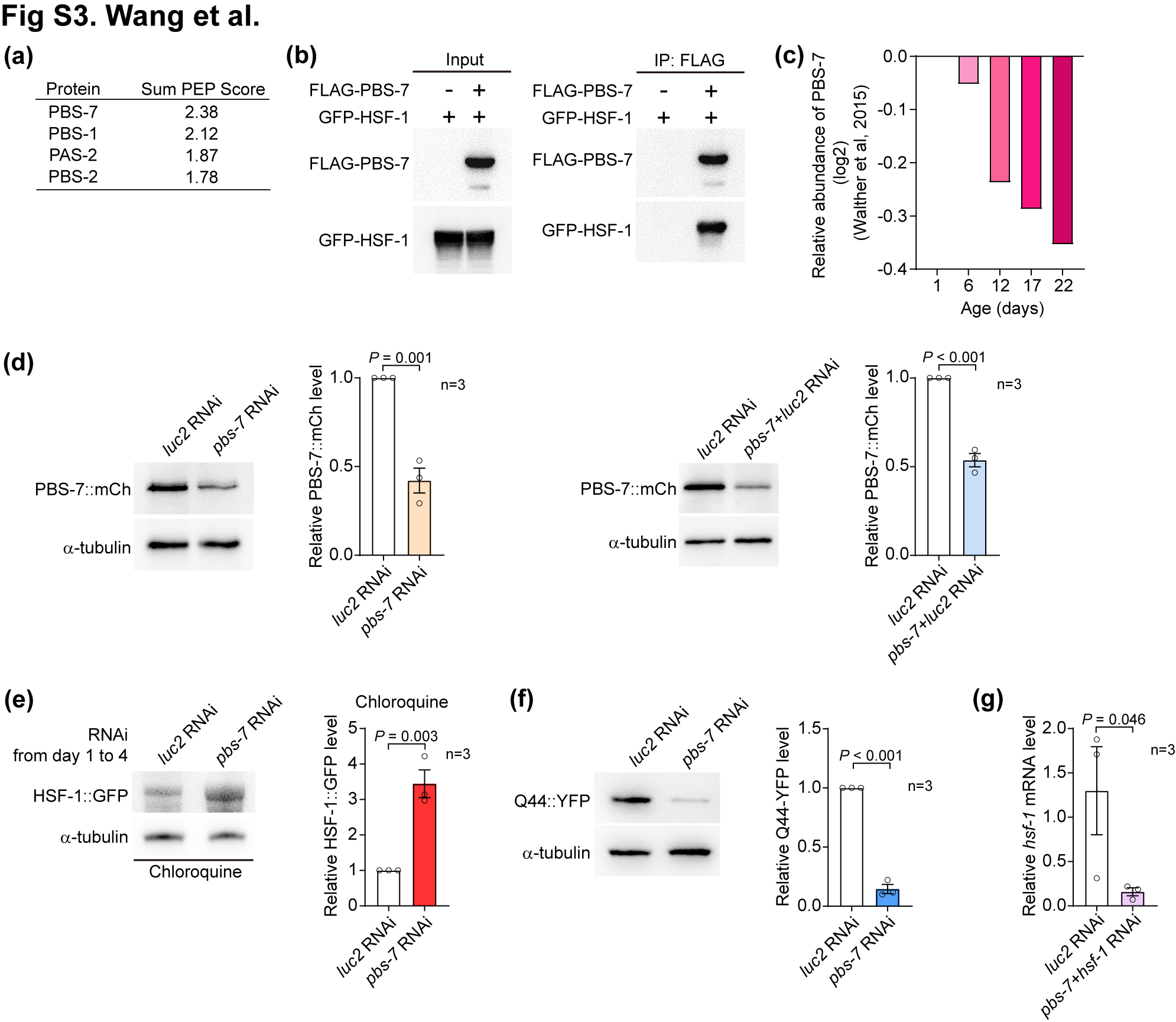

Supplement: Supplementary file 3 — Figure S3: The interaction between PBS‐7 and HSF‐1 decreases with ageing. (a) PBS‐7 shows the highest confidence as HSF‐1::GFP interactor among the four proteosome components specifically detected in HSF‐1::GFP immunoprecipitants of young worms. Sum PEP (posterior error probability) Score is a metric to assess the confidence of an identified protein. The higher it is, the higher the confidence. (b) The co‐immunoprecipitation of FLAG‐PBS‐7 with GFP‐HSF‐1 in HEK293T cells. Representative blots from 3 biological replicates were shown. (c) Quantitative proteomic analysis shows an age‐dependent decrease of PBS‐7. The level of PBS‐7 at Day 1 of adulthood is set as 1. Data are from Walther et al. 2015. (d) RNAi efficiency of indicated pbs‐7 RNAi treatments. PBS‐7::mCh is expressed from a single copy transgene, pbs‐7p::pbs‐7::mCh, inserted at ttTi5605 by CRISPR‐Cas9. Representative blots from 3 biological replicates were shown. (e) Chloroquine treatment does not block the upregulation of HSF‐1::GFP upon pbs‐7 RNAi in young worms. (f) The protein level of Q44::YFP upon indicated RNAi treatment. (g) hsf‐1 RNAi efficiently knocks down the mRNA levels of hsf‐1. α‐tubulin serves as the loading control in (d, e, and g). Unpaired t‐test. 3 biological replicates were examined in (b–g). Error bars: SEM. [file ACEL-25-e70399-s004.tif]

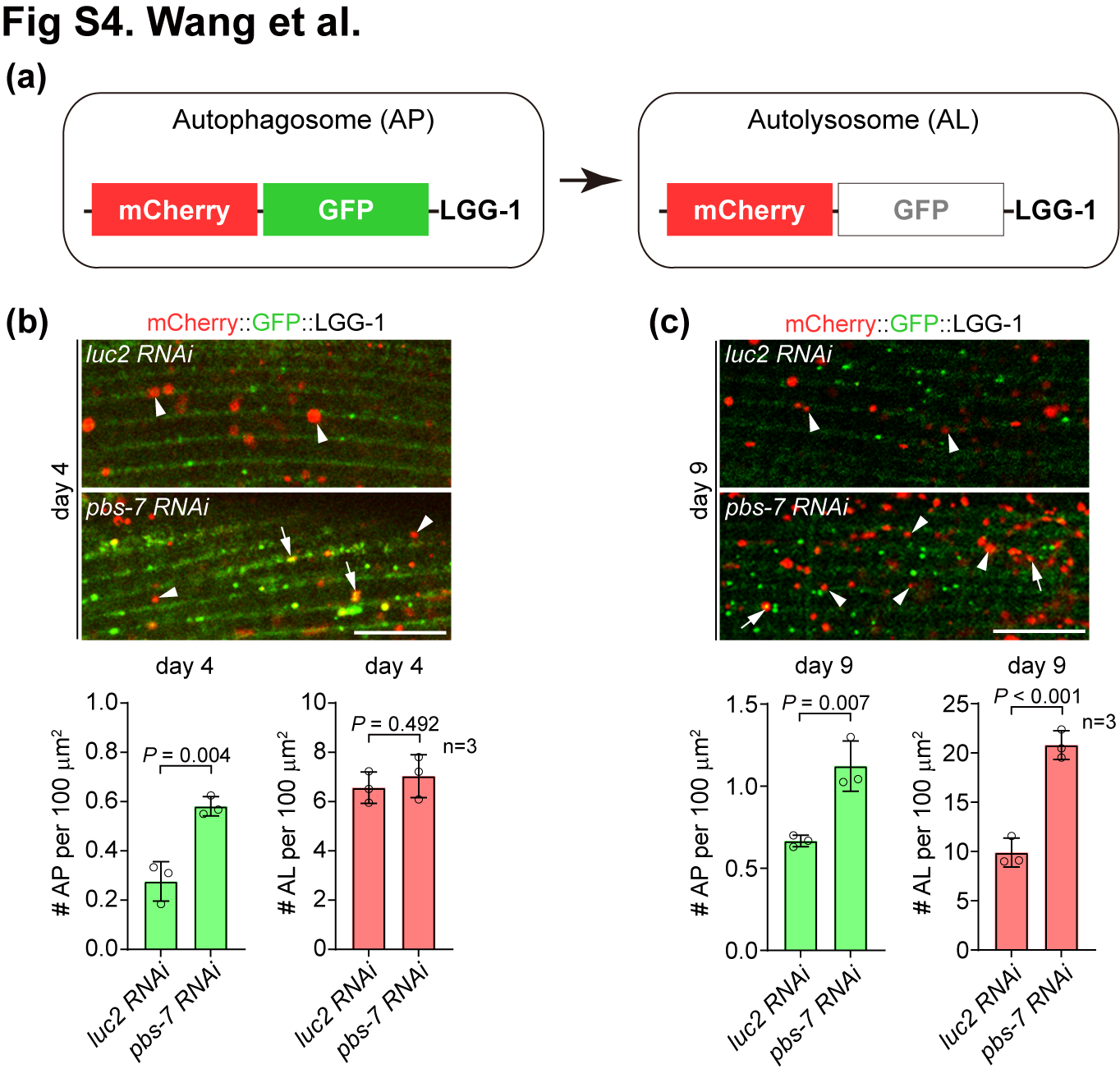

Supplement: Supplementary file 4 — Figure S4: Suppressing pbs‐7 improves autophagy in the body wall muscle. (a) A depiction of the analysis of autophagosome (AP) and autolysosome (AL) using the mCherry::GFP::LGG‐1 reporter. The acidic environment in AL quenches GFP fluorescence, turning the reporter into red. (b and c) Worms carrying the mCherry::GFP::LGG‐1 reporter were treated with indicated RNAi from Day 1 to 4 (b) or Day 9 (c) of adulthood. APs and ALs were scored in the body wall muscle. Arrows: AP, arrowheads: AL. Scale bars: 10 μm. Unpaired t‐test. More than 77 worms in three biological replicates were examined. [file ACEL-25-e70399-s007.tif]

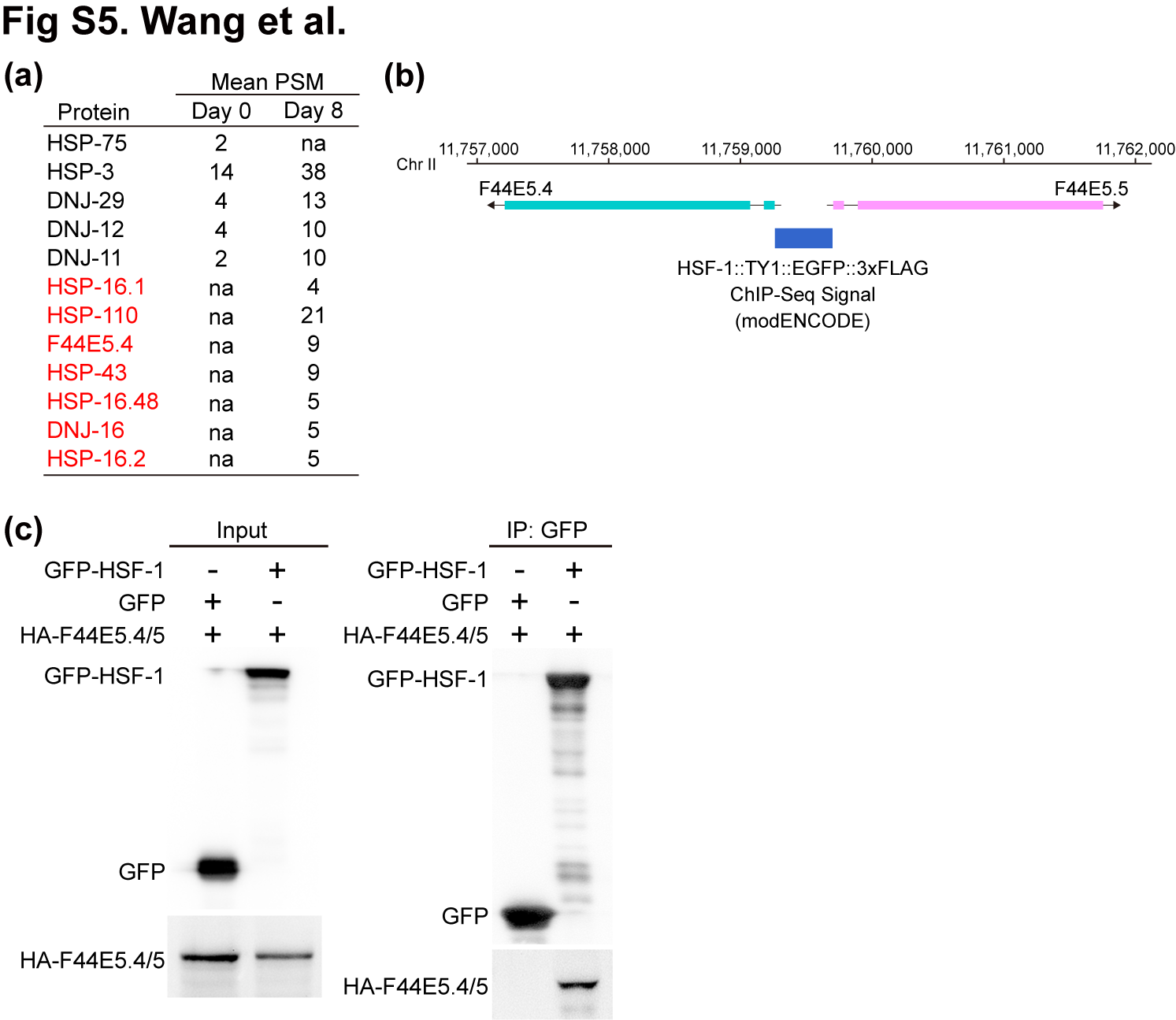

Supplement: Supplementary file 5 — Figure S5: The age‐dependent HSF‐1 interactions with heat shock proteins. (a) The detected heat shock proteins (HSPs) in the co‐immunoprecipitants of HSF‐1::GFP by mass‐spectrometry. PSM, peptide‐spectrum matches, was used as a benchmark for the interaction with HSF‐1::GFP. Those specifically found from worms at Day 8 of adulthood are highlighted in red. (b) A depiction of F44E5.4 and F44E5.5 in the genome. The two genes are identical in cDNA sequences and located closely on Chromosome II. By ChIP‐Seq datasets in modENCODE, HSF‐1 binds to their shared promoter region. (c) HA‐F44E5.4/5 interacts with GFP‐HSF‐1 in HEK293T cells. Representative blots from 3 biological replicates were shown. [file ACEL-25-e70399-s002.tif]

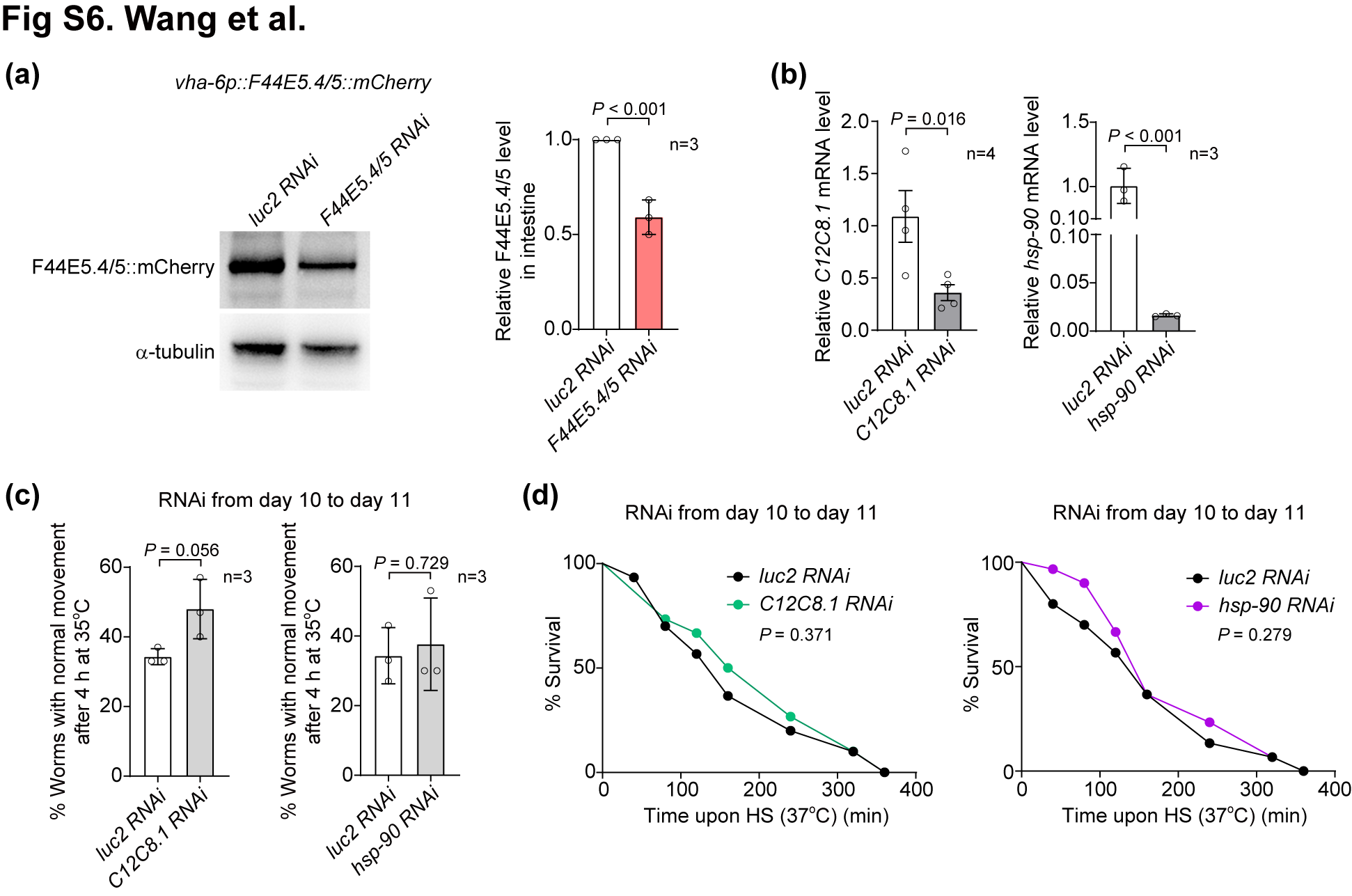

Supplement: Supplementary file 6 — Figure S6: RNAi against C12C8.1 or hsp‐90 in aged worms barely improved heat resistance. (a) RNAi efficiency of F44E5.4/5 RNAi. The worms expressing F44E5.4/5::mCherry in the intestine were treated with indicated RNAi and examined by western blot. α‐tubulin serves as the loading control. 3 biological replicates were examined. (b) RNAi against C12C8.1 and hsp‐90 efficiently suppressed target genes mRNA levels. 3 or 4 biological replicates were examined. (c and d) C12C8.1 or hsp‐90 RNAi in aged worms had little effect on worms recovery post 4‐h heat shock at 35°C (c) or the survival upon a 37°C heat shock (HS) (d). More than 60 worms in three biological replicates were examined. Unpaired t‐test in (a–c), Gehan‐Breslow‐Wilcoxon test in (d). [file ACEL-25-e70399-s001.tif]
